# Do pre-hospital poisoning deaths differ from in-hospital deaths? A retrospective analysis

**DOI:** 10.1186/s13049-017-0391-z

**Published:** 2017-05-08

**Authors:** Lauri Koskela, Lasse Raatiniemi, Håkon Kvåle Bakke, Tero Ala-Kokko, Janne Liisanantti

**Affiliations:** 1 Department of Anesthesiology, Division of Intensive Care Medicine, Oulu University Hospital, P.O. BOX 21, 90029 OYS Oulu, Finland; 2 Medical Research Center, Study Group of Surgery, Anesthesiology and Intensive Care, Oulu University, Oulu, Finland; 3 Centre for Pre-Hospital Emergency Care, Oulu University Hospital, Oulu, Finland; 4 Anesthesia and Critical Care Research Group, University of Tromsø, Tromsø, Norway; 5 Mo i Rana Hospital, Helgeland Hospital Trust, Mo i Rana, Norway

**Keywords:** Poisoning, Intoxication, Alcohol, Finland, Mixed poisoning

## Abstract

**Background:**

Most fatal poisonings occur outside the hospital and the victims found dead. The purpose of this study was to determine the general pattern and patient demographics of fatal poisonings in Northern Finland. In particular, we wanted to analyze differences between pre-hospital and in-hospital deaths.

**Methods:**

All fatal poisonings that occurred in Northern Finland in 2007–2011 were retrieved from the Cause of Death Registry provided by Statistics Finland. We noted the patient demographics, causal agents, and other characteristics of the poisoning events.

**Results:**

A total of 689 fatal poisonings occurred during the study period, of which only 42 (6.1%) reached the hospital alive. Those who died pre-hospital were significantly younger (50 vs. 56 years, *p* = 0.04) and more likely to be male (77% vs. 57%, *p* = 0.003). Cardiopulmonary resuscitation was attempted less often in pre-hospital cases (9.9% vs. 47.6%, *p* < 0.001). Ethanol was more frequently the main toxic agent in pre-hospital deaths (58.4% vs. 26.2%, *p* < 0.001), and multiple ingestions were more common (52.2% vs. 35.7%, *p* < 0.001) in pre-hospital deaths.

**Discussion:**

Most of the pre-hospital fatal poisoning victims are found dead and the majority of in-hospital victims are admitted to hospital in an already serious condition. According to results of this and former studies, prevention seems to be the most important factor in reducing deaths due to poisoning.

**Conclusions:**

The majority of poisoning-related deaths occur pre-hospital and are related to alcohol intoxication and multiple ingestions.

## Background

The outcome for hospitalized patients with acute poisoning is good with a mortality rate of less than 5% [1–4]. The reported risk factors for hospital mortality in poisoned patients include respiratory and renal failure and hypotension. However, in-hospital death of poisoned patients, especially those with acute poisoning with pharmaceutical product, is rare [5–7]. Most fatal poisonings occur on scene and victims are found dead [5, 8]. Fatal poisonings are more common in Finland than other Nordic countries despite a decrease in incidence over the last few years [5, 9]. A previous Finnish study investigated pre-hospital and in-hospital fatal poisonings but did not compare the clinical features of the victims [5].

More knowledge is needed in order to reduce the number of fatalities by preventing poisoning events and offering more effective emergency care. Therefore, we evaluated the causes of fatal poisonings in Northern Finland and compared the pattern of toxic agents in fatal pre-hospital and in-hospital poisonings. More specifically, we compared the agent of ingestion, intent, gender, and age.

## Methods

The present descriptive observational study was based on death certificate data from the Finnish Cause of Death Registry (FCDR) provided by Statistics Finland. The study protocol was approved by the Oulu University Hospital administration (Reference no. 110 12015), the Regional Ethics Committee (Reference no. 982013), and Statistics Finland (Reference no. TK53-1151-13).

### Study population

The study area consisted of the four northernmost counties in Northern Finland. The area had a population of 728,847 inhabitants in 2011. All deaths from poisoning that occurred within the study area between January 1, 2007, and December 31, 2011, were included. Poisoning was defined as fatalities for which the cause of death was registered as ICD-10 diagnosis codes X40-49, X60-69, X85-90, Y10-Y19, T36-T50, T51-T65, Y34, Y57, Y84 [10]. Deceased patients who resided outside Finland were not included in the study as the FCDR only has data on the population residing in Finland. Fatal poisonings were divided into two categories: pre-hospital and in-hospital deaths. Death was defined as pre-hospital if it occurred on scene or during transportation to the hospital, whereas deaths occurring during primary admission to the hospital were defined as in-hospital death.

### The data

The death certificates were retrieved for all victims. Medical records were also retrieved for those who died in-hospital. The collected data consisted of patient demographics, the location of death (on scene, during transportation, in-hospital, and other facilities such as jail), intention of intake (suicide, unintentional, treatment failure, or unclear intentions), toxic agents, and involvement of alcohol.

According to Finnish law, medicolegal autopsy is performed if the cause or suspected cause of death is a crime, accident, suicide, poisoning, or unknown [11]. The screenings for alcohol, pharmaceutical products and illicit drugs is conducted in autopsy, if poisoning event is considered possible [12]. In Finland, post-mortem forensic toxicological investigations are centralized at the Hjelt Institute, University of Helsinki.

### Statistical analysis

The statistical analysis was conducted using IBM SPSS Statistics 22 software (IBM SPSS Statistics for Windows, Version 22.0, Armonk, NY, USA). Categorical data are presented as number and percent. Summary measurements are presented as median values with 25th and 75th percentiles and were analyzed using Mann-Whitney’s non-parametric test. Categorical variables were analyzed using Pearson χ^2^.

## Results

### Demographics of fatal poisonings in Northern Finland

A total of 2,915 trauma or poisoning deaths occurred in the study area between January 1, 2001, and December 31, 2011, and 689 (23.6%) were fatal poisonings. Most of the deceased were found dead and CPR was attempted significantly less often in victims of pre-hospital deaths than those who died in-hospital (Table 1). The number of annual in-hospital deaths varied from 6 (4.1%) to 11 (8.3%). The victims who died during their hospital stay were more likely to be female and older than those who died pre-hospital.

**Table 1:** Characteristics of fatal poisonings in Northern Finland 2007-2011

	Overall	Pre-hospital deaths	In-hospital deaths	*P*-value
	*N* = 689	*N* = 647	*N* = 42	
Age, y	50 [39–58]	50 [39–58]	56 [42–70.5]	0.04
Male gender	523 (75.9)	499 (77.1)	24 (57.1)	0.003
Found dead	528 (76.6)	528 (81.6)	-	
CPR	84 (12.2)	64 (9.9)	20 (47.6)	<0.001
GCS at admission	-	-	3 [3–9]	
ICU admission	-	-	32 (76.2)	
ICU LOS, h	-	-	48 [2–72]	
Hospital LOS, d	-	-	2 [1–4]	
Location of poisoning				
Home or acquaintance	620 (90.0)	586 (90.6)	34 (81.0)	0.002
Public place	49 (7.1)	45 (7.0)	4 (9.5)	
Health facility	10 (1.5)	6 (0.9)	4 (9.5)	
Imprisonment	10 (1.5)	10 (1.5)	0 (0)	
Motivation				
Suicide	122 (17.7)	112 (17.3)	10 (23.8)	0.36
Unintentional intake	524 (76.1)	496 (76.7)	28 (66.7)	
Unclear intention	43 (6.2)	39 (6.0)	4 (9.5)	
Autopsy rate	689 (100)	647 (100)	42 (100)	

Data are presented as n (%) or median [25^th^–75^th^ percentile]. *CPR* cardiopulmonary resuscitation, *GCS* glasgow coma scale, *ICU* intensive care unit, *LOS* length of stay

### Location of poisonings

Most of the victims in both groups were found in the victim’s home (Table 1). Ninety-three of the 647 (14.4%) pre-hospital deaths occurred in a residence of an acquaintance, compared to 3 (7.1%) in-hospital deaths. More information is provided in Table 1.

### Substances in fatal poisonings

Ethanol was the most common poisoning agent and was involved overall as the main toxic agent or as a contributory agent in 487 of the 647 (75.3%) pre-hospital deaths and 19 of the 42 (45.2%) in-hospital deaths (*p* < 0.001). Ethanol was the causative agent significantly more often in pre-hospital deaths than in-hospital deaths. For the in-hospital group, the second most common agent was methanol (Table 2).

**Table 2:** The main toxic agents in 689 fatal poisonings in Northern Finland in 2007–2011

	Overall	Pre-hospital	In-hospital	*P*-value
	*N* = 689	*N* = 647	*N* = 42	
Ethanol	389 (56.5)	378 (58.4)	11 (26.2)	<0.001
Methanol	22 (3.2)	14 (2.2)	8 (19.0)	<0.001
Ethylene glycol	9 (1.3)	9 (1.4)	0 (0)	>0.05
Psychoactive agents	106 (15.4)	103 (15.9)	3 (7.1)	>0.05
Atypic neuroleptic	16 (2.3)	16 (2.5)	0 (0)	
Neuroleptic	16 (2.3)	15 (2.3)	1 (2.4)	
Benzodiazepine	26 (3.8)	26 (4.0)	0 (0)	
Antidepressants	48 (7.0)	46 (7.1)	2 (4.8)	
Antiepileptics	20 (2.9)	19 (2.9)	1 (2.4)	>0.05
Analgesics	99 (14.4)	91 (14.1)	8 (19.0)	>0.05
Opioid	89 (12.9)	85 (13.1)	4 (9.5)	
Paracetamol	10 (1.5)	6 (0.9)	4 (9.5)	
Other	35 (5.1)	26 (4.0)	9 (21.4)	<0.001
Insulin	9 (1.3)	7 (1.1)	2 (4.8)	
Cardiovascular	15 (2.2)	12 (1.9)	3 (7.1)	
Other pharmaceutical product	11 (1.6)	7 (1.1)	4 (9.5)	
Other non-pharmaceutical	9 (1.3)	7 (1.1)	2 (4.8)	

Data are presented as n (%)

Of opioids, tramadol was found as the primary toxic agent in 31 (4.5%) of the post-mortem forensic toxicological screenings, codeine in 27 (3.9%) and buprenorphine in 12 (1.7%). There were no illicit street drugs found among the primary toxic agents of fatal poisonings. As a secondary toxic agent, amphetamine was found in 11 (1.6%) fatalities and cannabinoid agent in 10 (1.5%), but apart from those findings there were no signs of illicit street drugs. Heroin wasn’t found in any of the fatalities.

In suicides, ethanol was the main agent in of the 15 of the 122 cases (12.3%) followed by amitriptyline (11 (9.0%)), codeine (10 (8.2%)) and insulin (8 (6.6%)).

Multiple ingestions occurred more often in pre-hospital deaths than in in-hospital deaths (52.2% vs. 35.7%, *p* < 0.001). Most common combination included in multiple ingestions was ethanol and diazepam (*N* = 50), and generally the most common combination was ethanol combined with benzodiazepine (*N* = 136) or antidepressant (*N* = 83). The most common combination in co-ingestions where ethanol wasn’t involved was tramadol and diazepam (*N* = 16).

## Discussion

To the best of our knowledge, this study is the first to compare characteristics of poisoning-related pre-hospital and in-hospital deaths. We found that the majority of cases were found dead on scene and that ethanol was the main causative agent, particularly in pre-hospital deaths. In addition, multiple ingestions were more common in pre-hospital deaths than in-hospital deaths.

A previous study in Finland also found that most of the deceased in poisoning-related deaths are found dead [5]. CPR was attempted in only 10% of pre-hospital deaths, which was approximately 13 pre-hospital CPR attempts in response to poisoning per year in the study area. Subsequently, nearly half of the patients who died in the hospital were resuscitated at the scene, indicating a very serious condition before arrival at the hospital. This finding in line with our previous study indicating that severe organ failure, including respiratory and circulatory failure, were risk factors for hospital death in patients with acute poisoning who are admitted to the ICU [6].

When taking into account all fatal poisonings in which multiple agents were detected, ethanol was involved in more than 75% of cases, which was in line with a Finnish study from the late 1990s focusing on hospitalized poisonings, reporting an ethanol involvement rate of nearly 70% [13]. However, our finding is higher than in a Norwegian series focusing on poisoning diagnostics, which reported an ethanol involvement rate of 35% [14]. Approximately one-third of the fatal poisonings were related to pharmaceutical products (38%). We did not find any deaths caused by illicit street drugs. In the present study, psychoactive pharmaceutical products were the main toxic agent in only 7% of in-hospital and 16% of pre-hospital deaths. According to the present results, the causes of pre-hospital deaths related to pharmaceutical products have similar characteristics as hospital survivors reported in studies from Norway reporting a predominance of opioid and psychoactive pharmaceutical drugs [1, 7]. In the present study, these agents were involved in nearly one-third of pre-hospital deaths and only every sixth in-hospital death.

### Limitations and strengths

The main limitation of the present study is the low number of in hospitals-deaths, which partly limited the possibilities for comparing poisoning patterns between pre-hospital and in-hospital deaths. In addition, the data available from hospital stays were limited because of the retrospective design of the study. Furthermore, the suicide rate may be underestimated in the present study, as the intention of ingestion cannot be determined reliably, even in medicolegal autopsy. Suicidal intent is recorded only if there is clinical evidence supporting this, such as good-bye letter. However, with an autopsy rate of 100%, we present the most reliable results that can be reached in this population. Due to the high quality of death certificates after medicolegal autopsy, the location of death was definitive, as well as the pre-hospital emergency critical care.

Considering that the coverage of the cause of death registry is nearly 100% [15], the gathered data should accurately reflect the quality of poisoning deaths in the source population.

### Clinical significance

According to the present results and previous literature, prevention seems to be the most important factor when aiming to reduce the number of fatal poisonings in Finland; a majority of the victims are not reached early enough and non-survivors who are hospitalized are poisoned by methanol or are severely ill. Though the proportion of deaths resulting from ethanol poisoning was high, access to psycho-pharmaceutical agents is important as well as the prevention of alcohol use. The most common place where victims were found or the poisoning occurred was a residence, and only a few of the poisonings occurred in locations where the event could have been prevented, including imprisonment or health care facilities. Most of the victims were found dead and the possibilities for decrease the rate of poisoning deaths with advances in pre-hospital emergency care or critical care are limited.

In light of the present results, the most effective way to decrease overall poisoning-associated mortality in the source population seems to be preventing alcohol intoxication. Recognizing existing risk populations for alcohol abuse is one step in preventing alcohol intoxication and fatalities [16, 17]. In Finland, fatal alcohol poisonings seem to peak around weekends and festive events; a 1% increase in alcohol sales increases the number of fatal alcohol intoxications by 0.4% [18]. On a societal level, policy changes, such as banning alcohol advertising, making alcohol more expensive, and reducing availability, have been proven to be effective in reducing harm caused by alcohol, whereas increasing education and information on the harm caused by alcohol has not been shown to have as great of an effect [19]. Furthermore, removing methanol-based antifreeze solutions from the market should be considered. Upon joining the European Union in 1995, previously prohibited antifreezes containing methanol became available for sale in Finland. This resulted in a remarkable increase in fatal methanol poisonings in subsequent years [20].

## Conclusions

The majority of poisoning-related deaths occur pre-hospital and are related to alcohol intoxication and multiple ingestions. Most of the deceased are found dead, and those admitted to hospital alive are severely ill. The rate of ethanol poisoning is 2-fold higher among pre-hospital deaths compared to in-hospital deaths, and multiple ingestions are more frequent among pre-hospital deaths.

## Abbreviations

CPR: Cardiopulmonary resuscitation
GCS: Glasgow coma scale
ICD: International classification of diseases
ICU: Intensive care unit
LOS: Length of stay
